# Supplementary material for: Ovo, an open-source ecosystem for de novo protein design
Source: Commun Biol. 2026 Jun 20;9:1336. doi: 10.1038/s42003-026-10497-1 (PMC13658209; doi:10.1038/s42003-026-10497-1)
Supplement: Supplementary file 1 [file 42003_2026_10497_MOESM1_ESM.pdf]

nature portfolio

Peer Review File

Ovo, an Open-Source Ecosystem for De Novo Protein Design

Corresponding Author: Dr Danny Bitton

This manuscript has been previously submitted at another journal. This document only contains information relating to versions considered at Communications Biology.

This file contains all reviewer reports in order by version, followed by all author rebuttals in order by version.

Version 0:

Reviewer comments:

Reviewer #1

(Remarks to the Author)
Summary:

Prihoda et al. present "Ovo, an Open-Source Exosystem for De Novo Protein Design" that can be deployed locally, on HPC systems, or in the cloud. At its core, ovo functions as a workflow orchestration framework built around a standardized data model. It integrates state-of-the-art and commonly used protein design tools into modular workflows, allowing for visualization, analysis, filtering, and downstream processing of design outputs. Pre-defined workflows are provided for scaffold design, binder design and binder diversification. They reflect established best practices. Ovo is accessible through both a graphical user interface (web app) and a command-line interface, making it suitable for users with varying levels of technical expertise. Beyond integrating existing workflows, the authors introduce the ProteinQC module, which facilitates assessment of designed proteins and relates them against reference datasets (e.g., evaluating whether properties such as isoelectric point fall within ranges observed for natural proteins), thereby supporting informed selection for downstream applications.

Overall impression:

The manuscript is rather descriptive but clearly and coherently presents the ovo software package. The codebase is hosted in a clean and well-organized GitHub repository and is accompanied by extensive documentation. The software itself offers limited conceptual novelty and is primarily an integration and orchestration effort. However, the level of engineering maturity and completeness is noteworthy. The work arrives at a timely moment, as the protein design community is rapidly expanding and increasingly in need of robust, interoperable tooling. I performed limited testing of the software (see below). Installation on a local computer and a basic scaffold design worked out of the box.

Comments to the authors:

The manuscript is clear and concisely written, and I have only minor comments.

1.
I tested ovo installation and a basic RFdiffusion scaffold design:
- Installation of the web app (using conda), following only the non-optional steps given https://ovo.dichlab.org/docs/user_guide/installation.html
- RFdiffusion Quickstart (https://ovo.dichlab.org/docs/rfdiffusion/quickstart.html)
- RFdiffusion scaffold design (10 designs) with PDB downloaded by ID: run locally on a Macbook (no GPU), including ProteinQC, Explorer and Refolding (interface analyser not applicable for this test)

Installation worked out of the box. The tool is very useful for protein design, even for experienced users. I particularly appreciated the ability to relaunch only part of the workflow (e.g. just refolding with different configs), or copy of the inputs of a previous run, as well as uploading your own existing designs for just the QC. The overall user experience was very positive.

2.
It appears that AlphaFold2 is run with a single input sequence without the full sequence search pipeline to generate an MSA. While this approach is common in the protein design field, it would be helpful to explicitly state this choice at the relevant points in the Methods section and point to the appropriate references (Frank et al., 2024?).

3.
The data availability statement refers to the Jupyter notebooks and the demo server hosting the examples described in the Methods section. If these projects are intended to remain persistently available on the demo server, it would be helpful to explicitly reference the corresponding OVO project IDs and the specific Jupyter notebooks used in each subsection. In addition, please verify the numerical values reported in the text. For example, in the RFdiffusion scaffold design case study on oxidoreductase motif scaffolding (starting from PDB 1A4I), the manuscript reports 13 accepted designs, whereas the corresponding OVO project (ogc I guess?) on the demo server lists 26.

4.
Maybe a small bug in the web app? When looking at the jobs in the demo server, the overview states excessive durations that keep getting longer even though the jobs are marked as done.

Reviewer #2

(Remarks to the Author)
This manuscript provides an overview of Ovo, an open-source protein design ecosystem that integrates existing published tools into reproducible workflows and a harmonized data model. Overall, it is a relatively mature open-source project that has been developed according to software development best practices. The manuscript provides comprehensive implementation detail, the code base is well documented with examples, and the project as a whole will lower the barrier of entry to protein design to newcomers. The research community will benefit from learning about this tool in a Comms Bio publication.

The only major technical feedback I have is:

1. The github repository does not currently have any continuous integration tests running. While I can see from inspecting the code base that there are comprehensive test cases, it would make my job as a reviewer much easier if they simply had their github repository configured to run automated tests.

Other than this, most of the following recommendations are small changes that might improve the clarity and impact of the narrative:

2. Given that the featured workflows (e.g., scaffold design, binder design, etc.) comprise a significant part of the narrative, a brief "teaser" mention in the Abstract and, additionally, a brief paragraph introducing/summarizing the workflows in the Introduction would orient the reader to expect the subsequent subsections in the Results.

3. For each of the design workflows and their respective protein targets (e.g., building a scaffold around an oxidoreductase active site, designing a binder to insulin receptor), provide some motivation and context describing how those targets represent a realistic drug/therapeutics/vaccine design task.

4. I suggest revising the subsection headers to improve the overall clarity:
a. First, be consistent with the structure of the header – e.g., if you use complete sentences, use complete sentences for all the headers; if you use short, concise phrases, do so for all the headers. (I prefer the latter, but leave it to the authors' judgment)
b. Consider including "Use case x" or "Workflow x" as a prefix in the respective headers—it might make the structure of the manuscript a little easier to understand when skimming the document. (I also leave this to the authors' judgment whether it works in practice)
c. Finally, many of the headers sound somewhat like language from a marketing brochure, either because they use subjective words like "intuitive" and "user-friendly" or because they repeatedly call out the name of the platform (Ovo, Ovo, Ovo...). Perhaps consider using more straightforward, objective-sounding headers.

5. Under the scaffold design subsection, the difference between the oxidoreductase and PD-1 use cases was not entirely clear. Are these use cases distinctly different from one another? And, if so, please include a short description of how these use cases contrast. If they aren't distinctly different, then is it necessary to include both?

6. In the binder design subsection, how do the BindCraft vs. RFDiffusion use cases compare and contrast? Please include a short, high-level description. Furthermore, the statement "by combining interface metrics, users may select designs with strong predicted binding, substantial interfaces, and low aggregation propensity" is currently buried almost as an afterthought, when it should be stated up front sooner to help motivate discussion of subsequent implementation details.

My final (optional) recommendation as a word of encouragement to the authors:

7. I would recommend that the authors consider teaching a tutorial session at the next International Workshop for Biodesign Automation (iwbdaconf.org), if they are able to make the journey.

Reviewer #3

(Remarks to the Author)
As interest in AI-based de novo protein design increases, numerous efforts are underway to design de novo proteins. Using computational design toolkits could be a bottleneck for those without prior computational experience. In this paper, the authors developed an open-source platform, Ovo, that integrates multiple software, manages data from protein design tools, analyzes the biochemical properties of designs, and visualizes the analysis results. Ovo is expected to help to lower the barrier for the protein design/engineering community to use computational methods. The authors convincingly showed how a user can use OVO to achieve their design goals. Ovo would be attractive for advanced users to customize their design pipelines by supporting external third-party Python packages and its own Ovo plugins. Exemplary application with miniprotein binder and oxidoreductase demonstrated the guide and usage of Ovo. However, I'd like to suggest a few crucial things to be improved for publication in Communications Biology.

1. I encourage the authors to include the open-source software for file format conversion (such as openbarbel, RDKit, biotite, etc.) and support easy file conversion in a GUI. Each user and software prefers to use different input and output formats to load or save structural information. Both pdb and cif format are commonly used for protein. In case of other chemical compounds, xyz, sif, mol, and mol2 are commonly used by the community.

2. Recently, Alphafold3 and its reproduced AI model (RosettaFold3, Chai-1, Boltz-2, Proteinix, etc.) achieved a significant improvement in the biomolecular interaction prediction task. The protein design and engineering community switched AlphaFold2 to AlphaFold3 as a design filter. I encourage authors to include AlphaFold3 or other reproduced models in Ovo.

3. A generative AI-based structure generation model has been extended to model atom-wise motif scaffolding. RFdiffusion2 and RFdiffusion3 are publicly available (https://github.com/RosettaCommons/foundry) at this moment. All-atom generative models have the capacity to generate more diverse functional proteins under user-defined constraints.

4. AF2 backpropagation-based optimization (BindCraft) is updated to AF3 backpropagation-based optimization (Boltzdesign1, https://github.com/yehlincho/BoltzDesign1). AF3 backpropagation-based method opened a new possibility for designing different types of biomolecular interactions. I urge to include AF3 backpropagation-based optimization method in Ovo, too.

5. Minor correction on Supplementary Table S4.OVO data model. The data type of 'contig_index' is needed to change from INTEGER to VARCHAR, as a manuscript mentioned "users select fixed residue segments and interactively specify a "contig" string that defines the order in which the fixed stretches should be connected (original order by default, but alternative permutations can be considered) and how many residues to design between them, and on N/C termini.".

Reviewer #4

(Remarks to the Author)
Summary

In this manuscript, the authors introduce OVO, an open-source ecosystem for de novo protein design. The platform integrates several widely used protein design tools (such as RFdiffusion, ProteinMPNN, AlphaFold2, PyRosetta and BindCraft) into a cohesive pipeline orchestrated by Nextflow. OVO is designed to be scalable, supporting execution environments ranging from local machines to High-Performance Computing (HPC) clusters and the AWS HealthOmics platform. The system features a web-based user interface (UI) that simplifies the specification of design parameters, management of design campaigns, and visualization of results. Additionally, a plugin mechanism allows for the integration of future design methods and filters.

Overall, OVO represents a useful engineering contribution that lowers the barrier to entry for running complex protein design workflows. However, I have several concerns regarding the system's flexibility, data accessibility, and error handling that should be addressed.

Major Comments

1. Verification of pipeline fidelity and performance overhead. As a pipeline paper rather than a method paper, relying on in silico results is acceptable. However, it is crucial to demonstrate that wrapping these tools in OVO does not introduce unexpected artifacts. The random noise plays a big role in those generative models. I would suggest that the authors verify that the OVO pipeline yields statistically similar results to running the underlying tools (RFdiffusion, ProteinMPNN, etc.) sequentially in their standalone versions. Additionally, the authors should quantify the computational overhead introduced by OVO. Is there a significant difference in runtime or resource usage compared to a "bare-metal" execution of the same scripts?

2. Support for customized workflows. While the pre-defined pipelines lower the entry barrier, they may limit advanced users who require customization. For instance, a common workflow involves identifying "good" backbones (e.g., those with at least one sequence passing AlphaFold filtering) and then resampling them more with ProteinMPNN using a higher temperature to

get more diverse sequences. The authors mention that OVO Python modules can be imported into Jupyter for downstream analysis. It would be highly beneficial if the authors provided a concrete Jupyter notebook example demonstrating how to perform this specific task: selecting a couple of promising backbones from an OVO run and triggering an extra MPNN-AF2 recycle loop.

3. Data organization and directory structure. The file storage structure under OVO_HOME appears to rely on Nextflow's hashed work directories (e.g., project_home/workdir/work/20/d86d1d...), which makes locating specific PDB files for external analysis difficult. Relying solely on the web UI to find files is restrictive. I suggest the authors expose the direct file paths in the web interface or implement a more human-readable directory structure for the final outputs (e.g., symlinking results to a named output folder). This would greatly facilitate downstream analysis outside of the OVO ecosystem.

4. Job status reporting. Currently, the "Job Status" indicator appears to show a generic Running state. Given that these pipelines involve distinct, time-consuming stages, this is insufficient for monitoring progress. I would suggest displaying the specific step of the pipeline (e.g., RFdiffusion, LigandMPNN, AlphaFold2) to provide a better user experience and help users estimate remaining time.

5. Resume failed tasks. Robust error recovery is essential for HPC-scale design campaigns. I encountered a specific issue where a job failed during a singularity image pull, and I was unable to resume the workflow from that point. It will be great if the authors could make OVO expose Nextflow's resume functionality or provide a clear UI mechanism to retry failed steps without manually resubmitting the entire campaign.

Note to authors: The error I encountered was java.lang.IllegalStateException: Failed to pull singularity image. While the server had internet access and I could pull the image manually. Could be a potential bug?

Minor Comments

1. Compared to ProteinDJ, the OVO web application significantly simplifies job creation. However, configuring the `nextflow.config` file for slurm remains a high barrier to entry. It would be a valuable addition if the web application included a slurm config to help users generate these config files for their specific compute environments.

Version 1:

Reviewer comments:

Reviewer #1

(Remarks to the Author)
Thanks for addressing my comments

Reviewer #2

(Remarks to the Author)
The authors have addressed my concerns, and I found this version of the manuscript to be a smooth, enjoyable, and educational read that benefited from the addition of interesting biological context for the example use cases, as well as other improvements in response to the other reviewers.

I especially appreciated this little bit, which paints a clear picture of the contrast between the binder-design use cases in a way that even an undergraduate biochemistry student would appreciate:

In contrast to the oxidoreductase example, which focuses on positioning a small set of catalytic residues within an enzyme core, the PD-1/PD-L1 example demonstrates the ability to maintain an extended protein-protein interaction motif in the precise three-dimensional geometry required for binding at the functional site

Neat!

I also appreciated that the authors addressed the recently released ProteinDJ pipeline. Their comparison of Ovo to ProteinDJ seemed fair and objective to me. I was also pleasantly amused by the following, which subverts any criticism of Ovo lacking any additional functionality that ProteinDJ may have:

To illustrate the extensibility of Ovo, we integrated the ProteinDJ pipeline as a plugin, which attests to how quickly community tools can be combined to expand the shared Ovo ecosystem.

(The big fish eats the little fish.)

I have a few minor remaining issues which I would ask the authors to consider, but I leave it to their judgement to address these, and do not require any additional review for my part.

• In lines 412, 414, and 423 the word “hallucinate” is used. Perhaps it would be better to use the word “generate”. In my experience, “hallucination” generally implies an undesirable side-effect of a model, so it may not be the term you want to use here.
• I found it curious in Fig. 3 that the insulin-receptor binder produced by BindCraft has three helices, while the RDdiffusion binder only has one, especially considering the “negative helicity loss” in BindCraft that the authors noted. I do not think this is an error, and likely most readers will not notice, but the authors should consider if they want to address this.

Thank you to the authors for their contribution. I look forward to using Ovo!

Reviewer #3

(Remarks to the Author)
In the revised version all my comments were addressed. No additional remarks.

Reviewer #4

(Remarks to the Author)
The authors solved all my concerns in the rebuttal. And I saw a lot of new commits in the repo. I am looking forward to v1.0.3!

Open Access This Peer Review File is licensed under a Creative Commons Attribution 4.0 International License, which permits use, sharing, adaptation, distribution and reproduction in any medium or format, as long as you give appropriate credit to the original author(s) and the source, provide a link to the Creative Commons license, and indicate if changes were made.
In cases where reviewers are anonymous, credit should be given to 'Anonymous Referee' and the source.
The images or other third party material in this Peer Review File are included in the article’s Creative Commons license, unless indicated otherwise in a credit line to the material. If material is not included in the article’s Creative Commons license and your intended use is not permitted by statutory regulation or exceeds the permitted use, you will need to obtain permission directly from the copyright holder.
To view a copy of this license, visit https://creativecommons.org/licenses/by/4.0/

Public

Response to Reviewer Comments

Thank you very much for taking the time to review this manuscript. Please find the detailed responses below and the corresponding revisions/corrections highlighted/in track changes in the re-submitted files.

Reviewer #1 (Remarks to the Author):

Summary:

Prihoda et al. present "Ovo, an Open-Source Exosystem for De Novo Protein Design" that can be deployed locally, on HPC systems, or in the cloud. At its core, ovo functions as a workflow orchestration framework built around a standardized data model. It integrates state-of-the-art and commonly used protein design tools into modular workflows, allowing for visualization, analysis, filtering, and downstream processing of design outputs. Pre-defined workflows are provided for scaffold design, binder design and binder diversification. They reflect established best practices. Ovo is accessible through both a graphical user interface (web app) and a command-line interface, making it suitable for users with varying levels of technical expertise. Beyond integrating existing workflows, the authors introduce the ProteinQC module, which facilitates assessment of designed proteins and relates them against reference datasets (e.g., evaluating whether properties such as isoelectric point fall within ranges observed for natural proteins), thereby supporting informed selection for downstream applications.

Overall impression:

The manuscript is rather descriptive but clearly and coherently presents the ovo software package. The codebase is hosted in a clean and well-organized GitHub repository and is accompanied by extensive documentation. The software itself offers limited conceptual novelty and is primarily an integration and orchestration effort. However, the level of engineering maturity and completeness is noteworthy. The work arrives at a timely moment, as the protein design community is rapidly expanding and increasingly in need of robust, interoperable tooling. I performed limited testing of the software (see below). Installation on a local computer and a basic scaffold design worked out of the box.

Comments to the authors:

The manuscript is clear and concisely written, and I have only minor comments.

1.
I tested ovo installation and a basic RFdiffusion scaffold design:
- Installation of the web app (using conda), following only the non-optional steps given https://ovo.dichlab.org/docs/user_guide/installation.html
- RFdiffusion Quickstart (https://ovo.dichlab.org/docs/rfdiffusion/quickstart.html)
- RFdiffusion scaffold design (10 designs) with PDB downloaded by ID: run locally on a Macbook (no GPU), including ProteinQC, Explorer and Refolding (interface analyser not applicable for this test)

Installation worked out of the box. The tool is very useful for protein design, even for experienced users. I particularly appreciated the ability to relaunch only part of the workflow (e.g. just refolding with different configs), or copy of the inputs of a previous run, as well as uploading your own existing designs for just the QC. The overall user experience was very positive.

Answer: We thank the reviewer for extensive testing and their positive comments about the tool and the installation process.

2.
It appears that AlphaFold2 is run with a single input sequence without the full sequence search pipeline to generate an MSA. While this approach is common in the protein design field, it would be helpful to explicitly state this choice at the relevant points in the Methods section and point to the appropriate references (Frank et al., 2024?).

Answer: We thank the reviewer for their suggestion. We updated the Methods section on page 18 as follows:

*"Lastly, each design is independently validated by AlphaFold2 initial guess **single-sequence mode without MSA**[15,19] to identify those with consistent and confident binder–target poses".*

3.
The data availability statement refers to the Jupyter notebooks and the demo server hosting the examples described in the Methods section. If these projects are intended to remain persistently available on the demo server, it would be helpful to explicitly reference the corresponding OVO project IDs and the specific Jupyter notebooks used in each subsection. In addition, please verify the numerical values reported in the text. For example, in the RFdiffusion scaffold design case study on oxidoreductase motif scaffolding (starting from PDB 1A4I), the manuscript reports 13 accepted designs, whereas the corresponding OVO project (ogc I guess?) on the demo server lists 26.

Answer: We thank the reviewer for the suggestion. We fixed the discrepancy in the Results section on page 37:

*"… resulting in **26 accepted designs** when RFdiffusion active site weights were employed."*

We also added the following paragraph to the Methods section page 38:

"Design structures and descriptors can be accessed through the "Motif scaffolding" and "Interface scaffolding" tabs in the demo server https://ovo.dichlab.org/demo/designs. Jupyter notebooks used for programmatic submission can be found at https://github.com/MSDLLCpapers/ovo-examples/tree/main/jupyter_notebooks_example."

4.
Maybe a small bug in the web app? When looking at the jobs in the demo server, the overview states excessive durations that keep getting longer even though the jobs are marked as done.

Answer: We thank the reviewer for reporting this bug. This was due to changes in nextflow log output. We implemented a bugfix which will be released as part of OVO 1.0.3.

Reviewer #2 (Remarks to the Author):

This manuscript provides an overview of Ovo, an open-source protein design ecosystem that integrates existing published tools into reproducible workflows and a harmonized data model. Overall, it is a relatively mature open-source project that has been developed according to software development best practices. The manuscript provides comprehensive implementation detail, the code base is well documented with examples, and the project as a whole will lower the barrier of entry to protein design to newcomers. The research community will benefit from learning about this tool in a Comms Bio publication.

Answer: We thank the reviewer for their thoughtful comments and their positive feedback.

The only major technical feedback I have is:

1. The github repository does not currently have any continuous integration tests running. While I can see from inspecting the code base that there are comprehensive test cases, it would make my job as a reviewer much easier if they simply had their github repository configured to run automated tests.

Answer: We thank the reviewer for the suggestion. Our GitHub repository does run continuous tests, although these are only unit tests focused on the general logic that can be tested without additional dependencies (containers). We did not enable continuous integration tests in GitHub due to large demands on resources (CPU/GPU, memory, network, ~100GB disk space). However, we added more instructions on running these integration tests to the OVO developer guide.

Other than this, most of the following recommendations are small changes that might improve the clarity and impact of the narrative:

2. Given that the featured workflows (e.g., scaffold design, binder design, etc.) comprise a significant part of the narrative, a brief "teaser" mention in the Abstract and, additionally, a brief paragraph introducing/summarizing the workflows in the Introduction would orient the reader to expect the subsequent subsections in the Results.

Public

Answer: We thank the reviewer for their suggestion. We added the following sentence to the Introduction section on page 3:

"Scaffold design (also called motif scaffolding) produces a highly redesigned protein based on an input structure and a contig specification that defines which regions to preserve and how to connect them with newly designed segments. Binder design generates protein-binding proteins or peptides based on the target protein's structure, the desired binder length, and optionally a specified binding hotspot on the target"

And modified the last paragraph of the Introduction on page 6:

*"… Ovo is powered by a robust, scalable, high-performance, modular and extensible backend foundation that features key models, pre-defined **scaffold design and binder design** workflows, and advanced filtering capabilities based on confidence scores and numerous protein descriptors…"*

The Abstract contains a brief mention of the two workflows, so we did not expand on this further in order to remain concise:

*"Ovo features Nextflow-based workflow orchestration, a storage layer, and both command-line and graphical interfaces that democratize **scaffold design, binder design and diversification**, and validation workflows."*

> *3. For each of the design workflows and their respective protein targets (e.g., building a scaffold around an oxidoreductase active site, designing a binder to insulin receptor), provide some motivation and context describing how those targets represent a realistic drug/therapeutics/vaccine design task.*

Answer: We thank the reviewer for this insightful suggestion. Our primary goal in selecting these targets was to reproduce representative benchmarks from the original RFdiffusion publication. We have now made this explicit in the main text and also included short motivation for each target as follows:

Page 9

"To demonstrate the capabilities of Ovo in scaffold design, we selected two examples adapted from the original RFdiffusion publication that represent two major classes of functional protein design problems: enzyme active-site scaffolding and protein–protein interaction motif scaffolding[11]. First, we demonstrated scaffold design on an oxidoreductase[11] (Figure 2a–c), one of the largest enzyme classes, which catalyze redox reactions central to energy metabolism, biosynthesis, detoxification and cellular redox homeostasis. The task of enzyme scaffolding requires constructing a de novo backbone that preserves the relative geometry of the catalytic residues while embedding them in a stable, well-packed, and designable protein fold. Second, we reproduced one of the RFdiffusion functional-motif scaffolding benchmarks of scaffolding the natural PD-1 binding interface to design binders to the therapeutically relevant cancer checkpoint protein PD-L1[11,36] (Figure 2d–e). In contrast to the oxidoreductase example, which focuses on positioning a small set of catalytic residues within an enzyme core, the PD-1/PD-L1 example demonstrates the ability to maintain an extended protein–protein interaction motif in the precise three-dimensional geometry

Public

required for binding at the functional site[11,36]. Workflow parameters and summary statistics are provided in the Methods and in Supplementary Tables S2 and S3."

Page 15

"Ovo provides bespoke end-to-end mini-protein and linear peptide design pipelines that generate binders to a user-specified target surface, featuring two literature-validated protocols based on RFdiffusion[11] and BindCraft[18]. RFdiffusion[11] uses a diffusion model fine-tuned on protein structure denoising, conditioned on the target structure, to generate de novo binder backbones, which are then subjected to sequence design and filtering. This typically requires generation of thousands of backbones to obtain a small number of high-quality hits. In contrast, BindCraft[18] operates directly in sequence-structure space, using AlphaFold2 multimer backpropagation to iteratively refine randomly initialized binder sequences and poses to satisfy structural and confidence-based objectives, and thus produces fewer but generally high-quality designs per run. BindCraft can be an order of magnitude more computationally efficient than RFdiffusion with respect to the number of in-silico accepted designs (confirmed by our example design task described below), while maintaining the same experimental success rates[18]. On the other hand, BindCraft is slightly more memory intensive because it uses multiple AlphaFold2 model weights concurrently. In terms of structural diversity, RFdiffusion binder designs were shown to be predominantly helical for some targets (with broader topologies accessible via alternative "beta" model weights), whereas BindCraft explicitly incorporates a "negative helicity loss" to mitigate generation purely alpha-helical binders and increase non-helical content[18]. On the other hand, RFdiffusion can explore diversity unbiased by the AlphaFold2 loss function.

To demonstrate binder design capabilities of Ovo, we selected the insulin receptor (IR) benchmark[11] . Insulin receptor is one of the five human cell-surface or extracellular targets that had previously been targeted using RFdiffusion and earlier Rosetta-based binder design methods, selected for their relevance as biological probes and potential therapeutics. In this example the binders are designed to insulin binding site on the receptor L1 domain."

4. I suggest revising the subsection headers to improve the overall clarity:
a. First, be consistent with the structure of the header – e.g., if you use complete sentences, use complete sentences for all the headers; if you use short, concise phrases, do so for all the headers. (I prefer the latter, but leave it to the authors' judgment)

> *Consider including "Use case x" or "Workflow x" as a prefix in the respective headers—it might make the structure of the manuscript a little easier to understand when skimming the document. (I also leave this to the authors' judgment whether it works in practice)*
>
> *Finally, many of the headers sound somewhat like language from a marketing brochure, either because they use subjective words like "intuitive" and "user-friendly" or because they repeatedly call out the name of the platform (Ovo, Ovo, Ovo...). Perhaps consider using more straightforward, objective-sounding headers.*

Answer: We thank the reviewer for their feedback. We updated the headings to be consistent and avoid repeating the name of the platform, as follows:

- Introduction
- Results
  - Ovo protein design ecosystem
  - Functional motif scaffolding of enzymes and binding interfaces
  - Miniprotein binder design and diversification
    - Binder design with RFdiffusion
    - Binder design with BindCraft
    - Binder diversification with RFdiffusion
  - ProteinQC characterizes designs in context of natural proteins
    - Stability descriptors reveal lower sequence entropy but high fitness
    - Charge and solubility analysis shows similarity to well-behaved natural proteins
    - Structure-related descriptors as proxies to packing, stability, and aggregation propensity
  - Ovo plugins – a call to the community
- Discussion
  - Ecosystem for protein design
  - From limitations to opportunities
- Methods
  - Application architecture
  - Database and data model
  - Pipeline definition and execution
  - User installation and setup
  - Web application
  - RFdiffusion scaffold design workflow
  - RFdiffusion binder design and diversification workflow
  - BindCraft binder design workflow
  - ProteinQC workflow
  - Community contribution through plugins

> *5. Under the scaffold design subsection, the difference between the oxidoreductase and PD-1 use cases was not entirely clear. Are these use cases distinctly different from one another? And, if so, please include a short description of how these use cases contrast. If they aren't distinctly different, then is it necessary to include both?*

Answer: We thank the reviewer for this helpful comment. We have added a new paragraph where we highlight the similarities and differences between the two scaffolding problems. Please refer to the answer to previous comment #3.

> *6. In the binder design subsection, how do the BindCraft vs. RFDiffusion use cases compare and contrast? Please include a short, high-level description. Furthermore, the statement "by combining interface metrics, users may select designs with strong predicted binding, substantial interfaces, and low aggregation propensity" is currently buried almost as an afterthought, when it should be stated up front sooner to help motivate discussion of subsequent implementation details.*

Answer: We thank the reviewer for their suggestion. We have added a paragraph which compares Bindcraft and RFdiffusion. Please refer to the answer to previous comment #3.

Public

My final (optional) recommendation as a word of encouragement to the authors:

7. I would recommend that the authors consider teaching a tutorial session at the next International Workshop for Biodesign Automation (iwbdaconf.org), if they are able to make the journey.

Answer: We thank the reviewer for their encouragement. We are not planning to join this year's edition due to travel budget constrains but will consider joining the future editions.

Reviewer #3 (Remarks to the Author):

As interest in AI-based de novo protein design increases, numerous efforts are underway to design de novo proteins. Using computational design toolkits could be a bottleneck for those without prior computational experience. In this paper, the authors developed an open-source platform, Ovo, that integrates multiple software, manages data from protein design tools, analyzes the biochemical properties of designs, and visualizes the analysis results. Ovo is expected to help to lower the barrier for the protein design/engineering community to use computational methods. The authors convincingly showed how a user can use OVO to achieve their design goals. Ovo would be attractive for advanced users to customize their design pipelines by supporting external third-party Python packages and its own Ovo plugins. Exemplary application with miniprotein binder and oxidoreductase demonstrated the guide and usage of Ovo. However, I'd like to suggest a few crucial things to be improved for publication in Communications Biology.

Answer: We thank the reviewer for their thoughtful comments and positive feedback.

1. I encourage the authors to include the open-source software for file format conversion (such as openbarbel, RDKit, biotite, etc.) and support easy file conversion in a GUI. Each user and software prefers to use different input and output formats to load or save structural information. Both pdb and cif format are commonly used for protein. In case of other chemical compounds, xyz, sif, mol, and mol2 are commonly used by the community.

Answer: We thank the reviewer for their suggestion. We are not planning to support handling small molecules in the near future, although the plugin functionality enables the community to expand into the small molecule space. For macromolecules, we added support for structure input in cif format, which will be released in 1.0.3. We are planning to switch from pdb to mmcif as a primary format for storing and transferring structures in the future.

2. Recently, Alphafold3 and its reproduced AI model (RosettaFold3, Chai-1, Boltz-2,

Proteinix, etc.) achieved a significant improvement in the biomolecular interaction prediction task. The protein design and engineering community switched AlphaFold2 to AlphaFold3 as a design filter. I encourage authors to include AlphaFold3 or other reproduced models in Ovo.

Answer: We thank the reviewer for their suggestion. We have implemented support for refolding using Boltz-2, the functionality will be available in version 1.0.3. We are not planning to add AlphaFold3 as a built-in module due to need for a commercial license. We are planning to continue investing into integrating new methods, but our main focus is on expanding the foundation that enables the community to contribute, for example by introducing a database model for target structures and their annotations (such as conservation or predicted binding sites). AlphaFold3 could be introduced as a community "plugin " – an independent python package that can be published separately from the core Ovo repository. We provide extensive documentation on plugin development at https://ovo.dichlab.org/docs/developer_guide/plugin_development.html.

3. A generative AI-based structure generation model has been extended to model atom-wise motif scaffolding. RFdiffusion2 and RFdiffusion3 are publicly available (https://github.com/RosettaCommons/foundry) at this moment. All-atom generative models have the capacity to generate more diverse functional proteins under user-defined constraints.

Answer: We thank the reviewer for their suggestion. We are exploring RFdiffusion3 and will consider it for the second version of the end-to-end pipeline. We are also adopting our existing pipeline to accept generated backbones from different backbone design methods, so that these can be introduced by the community via separate plugins. This functionality should be available in Ovo version 1.0.3 or 1.0.4 at the latest.

4. AF2 backpropagation-based optimization (BindCraft) is updated to AF3 backpropagation-based optimization (Boltzdesign1, https://github.com/yehlincho/BoltzDesign1). AF3 backpropagation-based method opened a new possibility for designing different types of biomolecular interactions. I urge to include AF3 backpropagation-based optimization method in Ovo, too.

Answer: We thank the reviewer for their suggestion. We are not planning to invest into integrating BoltzDesign in the near-term future as mentioned before additional tools and workflows could be added as plugins by the community.

5. Minor correction on Supplementary Table S4.OVO data model. The data type of 'contig_index' is needed to change from INTEGER to VARCHAR, as a manuscript mentioned "users select fixed residue segments and interactively specify a "contig" string that defines the order in which the fixed stretches should be connected (original order by default, but alternative permutations can be considered) and how many residues to design between them, and on N/C termini.".

Answer: We thank the reviewer for their comment. Naming this variable “contig_index” might have been misleading, a better name might have been “subtask_index” – this variable is used for cases where the workflow combines multiple contigs in one run, such as when submitting all permutations of segment orderings in scaffold design. The contig itself is indeed a string (varchar) but it is not stored as a column in the database but inside the Design.spec column as a JSON document, as well as in the JSON representation of the Workflow parameters.

Reviewer #4 (Remarks to the Author):

Summary

In this manuscript, the authors introduce OVO, an open-source ecosystem for de novo protein design. The platform integrates several widely used protein design tools (such as RFdiffusion, ProteinMPNN, AlphaFold2, PyRosetta and BindCraft) into a cohesive pipeline orchestrated by Nextflow. OVO is designed to be scalable, supporting execution environments ranging from local machines to High-Performance Computing (HPC) clusters and the AWS HealthOmics platform. The system features a web-based user interface (UI) that simplifies the specification of design parameters, management of design campaigns, and visualization of results. Additionally, a plugin mechanism allows for the integration of future design methods and filters.

Overall, OVO represents a useful engineering contribution that lowers the barrier to entry for running complex protein design workflows. However, I have several concerns regarding the system's flexibility, data accessibility, and error handling that should be addressed.

Answer: We thank the reviewer for their positive comments.

Major Comments

1. Verification of pipeline fidelity and performance overhead. As a pipeline paper rather than a method paper, relying on in silico results is acceptable. However, it is crucial to demonstrate that wrapping these tools in OVO does not introduce unexpected artifacts. The random noise plays a big role in those generative models. I would suggest that the authors verify that the OVO pipeline yields statistically similar results to running the underlying tools (RFdiffusion, ProteinMPNN, etc.) sequentially in their standalone versions. Additionally, the authors should quantify the computational overhead introduced by OVO. Is there a significant difference in runtime or resource usage compared to a "bare-metal" execution of the same scripts?

Answer: We thank the reviewer for their comment. We believe that the pipeline fidelity should not be impacted and any performance overheads should be negligible in context of the high computational complexity of the tools themselves. We address the individual concerns here as follows:

- Pipeline fidelity and performance overheads due to dependency management. Ovo provides two main options for dependency management:
    - Conda & pip (Ovo default) – An established standard in the field, recommended way of installing most of the tools as suggested by the authors. This option is equivalent to installing each tool manually with conda and pip.
    - Containers (Docker, Singularity) – Ovo provides pre-built containers for the x86 AMD architecture and CUDA 12-supported GPUs. Running these containers on such architectures should not result in any noticeable overhead since they are able to use the native instructions of the platform. Running on different architectures such as Mac M1+ (ARM) is not supported or might result in overhead, however such architectures are rarely used for large scale design campaigns. If needed, users can re-build and benchmark the containers on their hardware and consider this effort beyond the scope of this publication. We have not observed any difference between running through Conda or Singularity on our hardware.
- Performance overheads due to our Nextflow implementation of the pipeline. Compared to custom implementations such as bash scripts, our Nextflow implementation might incur additional IO overheads due to manipulating a lot of PDB files that could be avoided by custom formats such as the "silent file" format that stores many designs in a single file. We are investigating a standardized file transfer format that would avoid this overhead, potentially using a zip archive of cif files instead of a folder of pdb files.
- Performance overheads due to Ovo file processing. When the pipeline is finished, Ovo needs to copy all the designs (potentially up to hundreds of thousands of PDB files) from the working directories into the Ovo storage directory (or S3 bucket). This can be time consuming especially on shared filesystems such as NFS due to file creation overhead. We implemented an option to store the structures in a zip archive to avoid the NFS overhead, which will be released in version 1.0.3.
- Impact of batching on randomness and diversity. RFdiffusion, ProteinMPNN and BindCraft do not use a fixed random seed by default, so each execution will produce different designs. The user has the option to specify a fixed random seed for reproducible design campaigns. In such case, each batch receives the seed plus an incremental offset, to ensure that each batch generates a different set of designs, while producing identical (or near-identical due to differences in hardware) results across rerunning the pipeline. By default, no fixed random seed is used. This means that designing 10 batches of 100 designs should be equivalent to designing 1 batch of 1000 designs. To verify this, we analyzed the 10 batches of 100 designs from the "mmo" pool of 1000 insulin receptor binder backbone designs. Only the first of each four sequence designs was considered. Foldseek easy-search with default settings clustered the 1000 designs into 775 separate clusters, indicating high diversity. We further computed all-by-all pairwise similarity (sparse, due to pre-filtering) of the 1000x1000 designs with folseek easy-search, to find the most similar structures for each structure. For each design, we computed the TM-score to its best-matching hit. The median of these best TM-scores was 0.496, indicating that similar designs were rarely produced. Only 12/1000 designs had hits above 0.8 TM-score and no hits were found above 0.9

Public

TM-score. For context, the median TM-score between different ProteinMPNN + fastrelax cycles of the same backbone design was 0.972.

2. Support for customized workflows. While the pre-defined pipelines lower the entry barrier, they may limit advanced users who require customization. For instance, a common workflow involves identifying "good" backbones (e.g., those with at least one sequence passing AlphaFold filtering) and then resampling them more with ProteinMPNN using a higher temperature to get more diverse sequences. The authors mention that OVO Python modules can be imported into Jupyter for downstream analysis. It would be highly beneficial if the authors provided a concrete Jupyter notebook example demonstrating how to perform this specific task: selecting a couple of promising backbones from an OVO run and triggering an extra MPNN-AF2 recycle loop.

Answer: We thank the reviewer for their suggestion. Currently, producing additional sequence designs for the same backbones is possible but only for binder design, through the "Binder diversification" workflow. This workflow includes partial diffusion using RFdiffusion, with a default number of 15 timesteps, which can be reduced down to 1 timestep in order to avoid re-designing the backbone and minimize the RFdiffusion computational cost (although still present). The Jupyter notebook for submitting this workflow is available here: https://github.com/MSDLLCpapers/ovo-examples/blob/main/jupyter_notebooks_example/notebooks/04_rfdiffusion_binder_design/01c_ovo_submit_partial_diffusion.ipynb

To provide more flexibility, we implemented the ability to provide a custom folder of backbones to the rfdiff-end-to-end pipeline, which will be available in upcoming 1.0.3 release or the following 1.0.4 release at the latest.

3. Data organization and directory structure. The file storage structure under OVO_HOME appears to rely on Nextflow's hashed work directories (e.g., project_home/workdir/work/20/d86d1d...), which makes locating specific PDB files for external analysis difficult. Relying solely on the web UI to find files is restrictive. I suggest the authors expose the direct file paths in the web interface or implement a more human-readable directory structure for the final outputs (e.g., symlinking results to a named output folder). This would greatly facilitate downstream analysis outside of the OVO ecosystem.

Answer: We thank the reviewer for their feedback. Ovo design files are actually copied from the "workdir" to the "storage" directory, where they are organized by the project ID and the pool ID, and the structure type (rfdiffusion, alphafold, proteinmpnn, …). However, we agree that this location is currently not transparent enough to the user. We are implementing a "Project" page that will more details about the current project and also technical information such as path to the storage directory for that project.

4. Job status reporting. Currently, the "Job Status" indicator appears to show a generic Running state. Given that these pipelines involve distinct, time-consuming stages, this is insufficient for monitoring progress. I would suggest displaying the

specific step of the pipeline (e.g., RFdiffusion, LigandMPNN, AlphaFold2) to provide a better user experience and help users estimate remaining time.

Answer: We thank the reviewer for their feedback. We implemented more advanced monitoring of the design jobs, it will be available in the 1.0.3 version.

Status monitoring and log output of individual tasks:

All tasks

	task_id	native_id	name	duration_seconds	status	exit	submit	duration	realtime	%cpu	peak_
☑	0d/3844cb	44459	RFdiffusion (1)	70	COMPLETED	0	2026-03-06 14:51:47.430	1m 10s	1m 5s	-	-
☐	77/a48b0c	44674	BackboneMetrics (1)	17.9	COMPLETED	0	2026-03-06 14:52:57.752	17.9s	15s	-	-
☐	c0/f9e878	44913	LigandMpnn (1)	25.7	COMPLETED	0	2026-03-06 14:53:15.647	25.7s	23s	-	-
☐	13/eb5d9a	45216	ProteinQC:proteinQCSeqComposition (1)	13.4	COMPLETED	0	2026-03-06 14:53:41.359	13.4s	10s	-	-
☐	e4/2a150e	45217	PyRosettaInterfaceMetrics (1)	31.8	COMPLETED	0	2026-03-06 14:53:41.367	31.8s	29s	-	-
☐	88/7c45ac	45551	Refolding:AlphaFoldInitialGuess (1)	30.3	COMPLETED	0	2026-03-06 14:54:13.214	30.3s	28s	-	-

Task log | COMPLETED | RFdiffusion (1)

```
Working directory: /Users/prihodad/ovo/workdir/work/0d/3844cb9a6d484b9c8bf904a5ecfa5b
+ mkdir -p output
+ export HYDRA_FULL_ERROR=1
+ HYDRA_FULL_ERROR=1
+ [[ null == \n\u\l\l ]]
++ python -c 'import site; print(site.getsitepackages()[0])'
+ SITE=/Users/prihodad/ovo/workdir/work/conda/env-fa85553f425440ecd8f4fdc763417c2f/lib/python3.11/site-packages
+ [[ ! -d /Users/prihodad/ovo/workdir/work/conda/env-fa85553f425440ecd8f4fdc763417c2f/lib/python3.11/site-packages/RFdiffusi
+ ln -s /Users/prihodad/ovo/workdir/work/conda/env-fa85553f425440ecd8f4fdc763417c2f/lib/python3.11/site-packages ./lib
```

Execution timeline (after workflow completion):

Processes execution timeline

Launch time: 04 Mar 2026 17:11
Elapsed time: 46s
Legend: job wall time / memory usage (RAM)

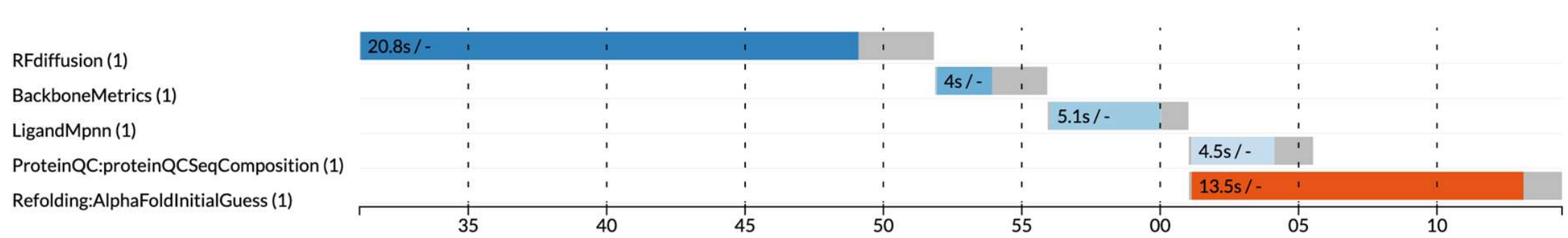

Workflow schema (after workflow completion):

Public

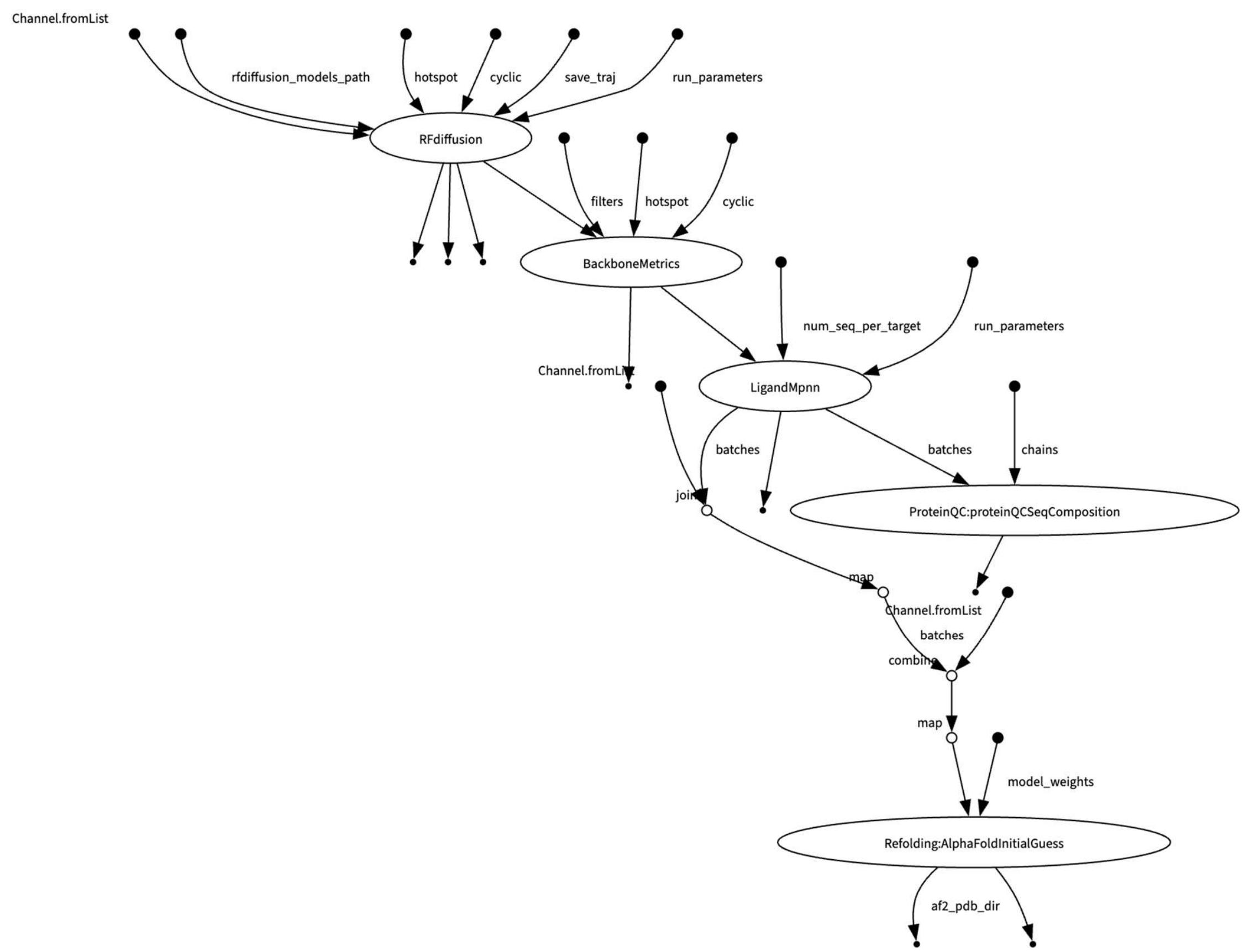

5. Resume failed tasks. Robust error recovery is essential for HPC-scale design campaigns. I encountered a specific issue where a job failed during a singularity image pull, and I was unable to resume the workflow from that point. It will be great if the authors could make OVO expose Nextflow's resume functionality or provide a clear UI mechanism to retry failed steps without manually resubmitting the entire campaign.

Answer: We thank the reviewer for their feedback. We implemented the ability to re-submit a failed workflow from the web interface using the nextflow resume functionality. This will be available in version 1.0.3.

Note to authors: The error I encountered was java.lang.IllegalStateException: Failed to pull singularity image. While the server had internet access and I could pull the image manually. Could be a potential bug?

Answer: We thank the reviewer for their report. This is a network issue, should be resolved by rerunning or increasing the image pull timeout.

Minor Comments

1. Compared to ProteinDJ, the OVO web application significantly simplifies job

creation. However, configuring the `nextflow.config` file for slurm remains a high barrier to entry. It would be a valuable addition if the web application included a slurm config to help users generate these config files for their specific compute environments.

Answer: We thank the reviewer for their feedback. We include an example slurm configuration file on the documentation page: *https://ovo.dichlab.org/docs/user_guide/schedulers.html#configuring-ovo-for-slurm-scheduler*. In case of further questions or suggestions, please feel free to submit an issue in our github repository: https://github.com/MSDLLCpapers/ovo/issues
