# Supplementary material for: Ovo, an open-source ecosystem for de novo protein design
Source: Commun Biol. 2026 Jun 20;9:1336. doi: 10.1038/s42003-026-10497-1 (PMC13658209; doi:10.1038/s42003-026-10497-1)
Supplement: Supplementary file 2 [file 42003_2026_10497_MOESM2_ESM.pdf]

Description of Additional Supplementary Files

File name: Supplementary Data 1

Description: The set of descriptors computed by Ovo's ProteinQC module

File name: Supplementary Data 2

Description: Statistics for motif scaffolding and binder design tasks

File name: Supplementary Data 3

Description: Selected ProteinQC and refolding descriptor values for 361 accepted designs (motif scaffolding, binder design, binder diversification)

File name: Supplementary Data 4

Description: OVO data model
